# Prevalence of Breech Presentation and Other Gestational/Delivery Characteristics Among Patients Born With Developmental Dysplasia of the Hip

**DOI:** 10.7759/cureus.42750

**Published:** 2023-07-31

**Authors:** Mofarreh M Khabiah, Aljohrah M. Al Hunaif, Enas A Al Mudawi, Khalid M Alkhalifah, Nouf A Asiri, Reemah F Alqahtani, Hajar A Alqahtani, Saud M Alzahrani

**Affiliations:** 1 Pediatric Orthopedics, Abha Maternity and Children Hospital, Abha, SAU; 2 College of Medicine, King Khalid University (KKU), Abha, SAU; 3 Unaizah College of Medicine and Medical Sciences, Qassim University, Ar-Rass, SAU; 4 Faculty of Medicine, King Khalid University (KKU), Abha, SAU; 5 College of Medicine, Umm Al-Qura University, Mecca, SAU

**Keywords:** newborns, congenital defect, prevalence, breech presentation, developmental dysplasia of the hip ( ddh )

## Abstract

Introduction

Developmental dysplasia of the hip (DDH) is the most common congenital disability in newborns. The condition can range from a slight laxity in the hip joint to secondary femoral head injury, early osteoarthritis (OA), and mobility issues. There are several risk factors for DDH, including positive family history, female sex, breech presentation, and the presence of clubfoot. Early detection and treatment are crucial to avoid long-term hip dysplasia and arthritis, which can cause difficulty in walking and discomfort. Breech presentation, in particular, is a significant risk factor for DDH, with spontaneous vaginal birth increasing the risk of hip pathology and instability compared to elective Caesarean section. However, whether breech presentation continues to be a risk factor for DDH in preterm children is unknown.

Objective and methods

This study aimed to investigate the prevalence of breech presentation and other gestational/delivery characteristics among newborns diagnosed with DDH. This retrospective study was conducted at Abha Maternity and Children Hospital, Abha, Saudi Arabia, over a period of six months. Data were collected from medical records of DDH cases diagnosed between 2016 and 2023. Data analysis was performed using Microsoft Excel (Microsoft Corporation, Redmond, Washington, United States) and IBM SPSS Statistics for Windows, Version 22 (Released 2013; IBM Corp., Armonk, New York, United States). Descriptive statistics and statistical tests were used to analyze the data.

Results

Most of the diagnosed children were female (86.7%), and bilateral hip dislocation (40%) was the most common presentation. X-ray was the most common diagnostic tool (48.3%), and operative management was the most common management strategy (73.3%). A positive family history of DDH was reported in more than one-third of cases. The study also analyzed the association between complications during pregnancy and various factors such as mode of delivery, presentation at delivery, gestational age at delivery, and associated maternal diseases. The difference in complication rates between women who delivered via C-section and those who delivered vaginally was insignificant (p = 0.14). Similarly, the difference in complication rates between women with breech and cephalic presentation was not statistically significant (p = 0.094). The difference in complication rates between women who delivered preterm, at term, or post-term was also not statistically significant (p = 0.578). Furthermore, the association between complications during pregnancy and pregnancy-associated maternal diseases was not statistically significant (p = 1.00).

Conclusion

DDH is a significant health issue in newborns, leading to long-term mobility problems and discomfort. Positive family history of DDH is a significant risk factor. Breech presentation was not significantly associated with DDH in preterm children, and no significant associations were found between complications during pregnancy and various factors. Early detection and treatment of DDH are crucial for preventing long-term complications. Family history should be considered an important risk factor, emphasizing the need for screening programs in families with a history of DDH.

## Introduction

Developmental dysplasia of the hip (DDH) is a complicated and divisive topic. It describes a range of hip issues, from stable hips with minor acetabular dysplasia to more serious types with outright hip dislocation. Early detection allows for effective treatment using less complex methods that are easily accessible, acceptable to the family, and have a good prognosis [1]. Positive family history, joint laxity, breech birth, being the first-born child, oligohydramnios, baby swaddling, female gender, multiple pregnancies, ethnicity, and the presence of various orthopedic conditions in infants, such as torticollis and foot deformities, have all been believed to be causes of or coexisting conditions with DDH and have frequently been mentioned as risk factors in the classical knowledge [2].

DDH causes various aberrations in the components that make up the hip joint, including anomalies in the femoral head socket and laxity in the supporting ligaments. It encompasses a variety of morphological anomalies and functional impairments that result from them. These conditions can manifest as slight laxity in the hip joint capsule or lead to secondary femoral head injury, early osteoarthritis (OA), and mobility issues [3]. DDH is the most common congenital defect in newborns, and its prevalence varies depending on whether population screening has been conducted and how it was performed (method and timing) [4]. Screening programs for DDH typically involve clinical evaluation during the neonatal period and well-child checkups, along with the use of ultrasound examination (either universal or selective) or a combination of both [5]. Moreover, some screening recommendations include conducting an ultrasonogram if the baby had a breech presentation in the third trimester of pregnancy [6]. Regardless of gestational age at delivery, the American Academy of Pediatrics (AAP) advises DDH surveillance with periodic physical examinations, further hip ultrasounds, or radiography after four months of age for newborns with recognized risk factors [5].

A previous meta-analysis study found that a positive family history of DDH, breech presentation, and female sex are important risk factors for DDH [4]. And these are all recognized as risks necessitating ultrasonographic evaluation in the United Kingdom. In Germany, Austria, and Switzerland, it is common practice to use ultrasound imaging to check all neonates for DDH at the time of birth [7]. The presence of a clubfoot also was associated with a greater risk of DDH [8]. Early detection and treatment can avert hip replacements due to long-term hip dysplasia and arthritis, which can cause complaints including difficulty in walking and hip, knee, and lower back discomfort [6]. The anatomy of the hip joint develops normally in utero, and hence there is no known etiology for DDH. Instead of being a congenital malformation, DDH is a developmental disorder [8].

Breech presentation is a significant risk factor for DDH, presumably due to the fetus being crammed inside the uterus. Because babies in multifetal pregnancies tend to be smaller than those in singleton pregnancies, the impact of breech presentation on fetal hips may be less obvious [9]. Compared to elective Cesarean section (C-section), spontaneous vaginal birth of breech newborns increases the risk of hip pathology and instability. However, the incidence of DDH was twice as high when vaginal birth was contrasted with an emergency C-section [8,10].

Breech presentation, which results in extended knee extension in utero and sustained hamstring pressures on the hip, is regarded as being at "high risk" for DDH. It is unknown whether breech presentation continues to be a risk factor for DDH in preterm children because they are exposed to intrauterine forces less frequently and for a shorter period of time [10,11]. 

In this retrospective study, we aimed to determine the prevalence of breech presentation and other gestational/delivery characteristics among newborns born with DDH.

## Materials and methods

The study was a single-center record-based cross-sectional analytic study conducted at the Abha Maternity and Children Hospital (AMCH) in Abha, Saudi Arabia. The target population in this study comprised all children diagnosed with DDH from January 2016 to March 2023. DDH patients were identified and their records were reviewed. A total of 155 participants were included in the study to estimate the prevalence of breech presentation and other gestational/delivery characteristics among patients born with DDH.

Study setting 

This study was conducted in the AMCH, one of the secondary hospitals in the Aseer region.

Sample size and participants

The total number of patients diagnosed with DDH in the AMCH between January 2016 and March 2023 was 260. After calculating the sample size using the EPI info program with a 95% confidence interval and a 5% margin of error, the estimated sample size was 155 patients. However, during the study, we were able to review 120 patient files.

DDH Screening Program in AMCH

AMCH is an orthopedic clinic that receives newborns from day 1 and onward, especially those who have risk factors such as breech presentation or a positive family history of DDH, or those who show suspicions of DDH based on routine Ortolani and Barlow tests performed by neonatologists immediately after delivery. Subsequently, newborns are clinically and radiologically evaluated by pediatric orthopedic surgeons. Usually, neonatal ultrasound for hips is conducted between the ages of four to six weeks until the age of three to four months. If a baby comes in after the age of three to four months and clinical examination suggests further evaluation through X-rays, it will be requested accordingly, and the appropriate management will be initiated.

Data collection 

Data collection was performed by manually extracting information from the patient's medical records using a data collection sheet. The sheet included data on the patient's gender, dislocation site, any complications during pregnancy (e.g., oligohydramnios), mode of delivery, presentation during delivery, any complications during delivery, gestational age at delivery, diagnosis method, management, associated maternal diseases, serial in the family, positive family history of DDH, the nature of pregnancy, and any other deformities. The study was conducted over a period of six months, which involved two months of data collection, two months of data analysis, and manuscript writing.

Statistical analysis

Microsoft Excel (Microsoft Corporation, Redmond, Washington, United States) and IBM SPSS Statistics for Windows, Version 22 (Released 2013; IBM Corp., Armonk, New York, United States) were used for data entry and analysis. Numerical data, such as age, were presented as mean ± standard deviation (SD). Categorical data were presented as frequencies and percentages. The main outcome of patients was presented as relative risks and 95% confidence intervals. Additionally, logistic regression was employed to identify significant risk factors. A test with a p-value less than 0.05 was considered statistically significant.

Ethical considerations

Ethical approval for this study was obtained from the Institutional Research Ethics Committee of the Ministry of Health at Asser region prior to the conduction of the study. Confidentiality and privacy were guaranteed. A de-identification coding system was used to protect patient information.

## Results

According to Table 1, which presents the sociodemographic and clinical parameters of children diagnosed with DDH, 86.7% of the diagnosed children were female, while 13.3% were male. In terms of birth order, 30.8% were first-born children, 17.5% were second-born, 19.2% were third-born, and 29.2% were born later in the family, with an additional 3.3% having an unknown birth order. The majority of the children (40.0%) had bilateral dislocation of the hip, while 35.8% had left-sided dislocation and 24.2% had right-sided dislocation. Clinical diagnosis was used in 30.8% of cases, while X-ray was used in 48.3%, ultrasound in 19.2%, and in 1.7% of cases, the method was unknown. Management of DDH was primarily operative (73.3%), with 15.8% managed non-operatively, 9.2% managed with both methods, and 0.8% received no management. Finally, regarding family history, 59.2% had no positive history of DDH, 34.2% had a positive history, and 6.7% had an unknown history.

**Table 1:** Sociodemographic and Clinical Parameters of Children Diagnosed With DDH DDH, developmental dysplasia of the hip; US, ultrasound.

		Frequency (n)	Percentage (%)
Gender	Female	104	86.7
	Male	16	13.3
Serial in the family	1st born	37	30.8
	2nd born	21	17.5
	3rd born	23	19.2
	Other	35	29.2
	Unknown	4	3.3
Dislocation site	Bilateral	48	40.0
	Left	43	35.8
	Right	29	24.2
Diagnosis method	Clinical	37	30.8
	Unknown	2	1.7
	US	23	19.2
	X-ray	58	48.3
Management of DDH	Operative	88	73.3
	nonoperative	19	15.8
	Both	11	9.2
	No management	1	0.8
	Unknown	1	0.8
Positive family history of DDH	No	71	59.2
	Yes	41	34.2
	Unknown	8	6.7
Total		120	100.0

Table 2 presents the gestational and delivery parameters of children diagnosed with DDH and their mothers. Of the 120 children, 119 (99.2%) had a normal nature of pregnancy, while the remaining 0.8% had an unknown pregnancy nature. Complications during pregnancy were present in 14.2% of cases, with the most common being oligohydramnios (6.7%). During delivery, 43.3% of children were delivered via C-section, while 56.7% were delivered vaginally. The breech presentation was present in 30.0% of cases, while 65.0% presented cephalic, and 1.7% presented transverse. Complications during delivery were present in 13.3% of cases, with delayed placental delivery being the most common (1.7%). In terms of maternal diseases, 6.7% of mothers had endocrine diseases, 3.3% had other diseases, and 89.2% had no associated diseases. The most common maternal disease was hypothyroidism (2.5%).

**Table 2:** Gestational and Delivery Parameters of Children Diagnosed With DDH and Their Mothers DVT, deep vein thrombosis; HTN, hypertension; GDM, gestational diabetes mellitus; DDH, developmental dysplasia of the hip; RA, rheumatoid arthritis.

		Frequency (n)	Percentage (%)
Nature of pregnancy	Normal	119	99.2
	Unknown	1	0.8
Any complications during pregnancy (e.g., oligohydramnios)	No	101	84.2
	Unknown	2	1.7
	Yes	17	14.2
Type of complication during pregnancy	None/Unknown	107	89.2
	Anemia	1	0.8
	Anhydramnios, placental abruption	1	0.8
	DVT in the left leg, pregnancy-induced HTN	1	0.8
	Fetal bradycardia	1	0.8
	GDM	1	0.8
	Oligohydramnios	8	6.7
Mode of delivery	C-section	52	43.3
	Vaginal	68	56.7
Presentation during the delivery	Breech	36	30.0
	Cephalic	78	65.0
	Transverse	2	1.7
	Unknown	4	3.3
Complications during delivery	Yes	16	13.3
	No	103	85.8
	Unknown	1	0.8
Type of complication during delivery	None/unknown	111	92.5
	Delayed placental delivery necessitating C-section procedure for the mother	1	0.8
	Fetal bradycardia	1	0.8
	Hemorrhage	1	0.8
	Labor dystocia, fetal distress, and emergency C-section	1	0.8
	Muscles spasm in the hands	1	0.8
	The cervix closed while there are strong contractions	1	0.8
	Uterine prolapse	1	0.8
	Umbilical cord wrapping	1	0.8
	Unknown	1	0.8
Gestational age at delivery	Post-term	7	5.8
	Preterm	15	12.5
	Term	98	81.7
Associated maternal diseases	Endocrine diseases	8	6.7
	No	107	89.2
	Other	4	3.3
	Unknown	1	0.8
Type of associated maternal disease	Unknown or no associated maternal disease	112	93.3
	Asthma	1	0.8
	DDH	1	0.8
	Hypothyroidism	3	2.5
	Leukemia	1	0.8
	RA	1	0.8
	Thyroid disease, anemia	1	0.8
Total		120	100.0

In conclusion, this study provides insights into the characteristics of infants with DDH in an urban setting. Females were more commonly affected, and bilateral dislocation was the most common presentation. X-ray was the most common diagnostic tool, and operative management was the most common management strategy. A positive family history of DDH was reported in more than one-third of cases.

The association between complications during delivery and various factors such as mode of delivery, presentation at delivery, gestational age at delivery, and associated maternal diseases was also analyzed using Fischer’s exact test. The results are presented in Table 3. Of the total 120 women who participated in the study, 103 (85.8%) had no complications during delivery, one (0.8%) had an unknown status, and 16 (13.3%) experienced complications.

**Table 3:** Association of Complication During Delivery With Mode of Delivery, Presentation at Delivery, Gestational Age at Delivery, and Associated Maternal Diseases (Fischer’s Exact Test)

		Any Complications During Delivery						
		No		Unknown		Yes		p-value
		Frequency (n)	Percentage (%)	Frequency (n)	Percentage (%)	Frequency (n)	Percentage (%)	
Mode of delivery	C-section	42	80.8	0	0.0	10	19.2	0.140
	Vaginal	61	89.7	1	1.5	6	8.8	
Presentation during delivery	Breech	31	86.1	0	0.0	5	13.9	0.094
	Cephalic	68	87.2	0	0.0	10	12.8	
	Transverse	2	100.0	0	0.0	0	0.0	
	Unknown	2	50.0	1	25.0	1	25.0	
Gestational age at delivery	Post-term	7	100.0	0	0.0	0	0.0	0.578
	Preterm	12	80.0	0	0.0	3	20.0	
	Term	84	85.7	1	1.0	13	13.3	
Associated maternal diseases	Endocrine diseases	7	87.5	0	0.0	1	12.5	1.000
	No	91	85.0	1	0.9	15	14.0	
	Other	4	100.0	0	0.0	0	0.0	
	Unknown	1	100.0	0	0.0	0	0.0	
	Total	103	85.8	1	0.8	16	13.3	

The analysis of the mode of delivery revealed that among the women who delivered via C-section, 80.8% had no complications, and 19.2% had complications. Among women who delivered vaginally, 89.7% had no complications, 1.5% had an unknown status, and 8.8% experienced complications. The difference in complication rates between the two modes of delivery was not statistically significant (p = 0.140).

Regarding presentation during delivery, 86.1% of women with breech presentation had no complications and 13.9% experienced complications. Among women with a cephalic presentation, 87.2% had no complications and 12.8% experienced complications. The difference in complication rates between the two groups was not statistically significant (p = 0.094).

In terms of gestational age at delivery, 7 (100.0%) of the women who delivered post-term had no complications. Among women who delivered preterm, 80.0% had no complications and 20.0% experienced complications. Among women who delivered at term, 85.7% had no complications, 1.0% had an unknown status, and 13.3% experienced complications. The difference in complication rates between the three groups was not statistically significant (p = 0.578).

Regarding associated maternal diseases, seven (87.5%) of the women with endocrine diseases had no complications and one (12.5%) experienced complications. Among women with no maternal diseases, 91 (85.0%) had no complications, 1 (0.9%) had an unknown status, and 15 (14.0%) experienced complications. The difference in complication rates between the groups was not statistically significant (p = 1.000).

Similarly, the association between complications during pregnancy and pregnancy-associated maternal diseases was also examined using Fischer’s exact test, and the results are presented in Table 4. Among those women who had endocrine diseases, 3 (37.5%) experienced complications during pregnancy, while among those who had no associated maternal diseases, 12 (11.2%) experienced complications during pregnancy. But this difference was found insignificant (p = 0.093). Among those who had other associated maternal diseases, two (50.0%) experienced complications during pregnancy. The woman with unknown maternal disease did not experience any complications.

**Table 4:** Association of Complication During Pregnancy With Pregnancy Associated Maternal Diseases (Fischer’s Exact Test)

		Any Complications During Pregnancy (e.g., Oligohydramnios)						
		No		Unknown		Yes		p-value
		Frequency (n)	Percentage (%)	Frequency (n)	Percentage (%)	Frequency (n)	Percentage (%)	
Associated maternal diseases	Endocrine diseases	5	62.5	0	0.0	3	37.5	0.093
	No	93	86.9	2	1.9	12	11.2	
	Other	2	50.0	0	0.0	2	50.0	
	Unknown	1	100.0	0	0.0	0	0.0	

In conclusion, the study found no statistically significant association between mode of delivery, presentation at delivery, gestational age at delivery, associated maternal diseases, and complications during pregnancy or delivery. However, the small sample size may limit the generalizability of these findings. Further research with a larger sample size is needed to confirm these results.

## Discussion

Our study found that the majority of the diagnosed children were female, with bilateral dislocation being the most common presentation. Additionally, operative management was the most common approach, and the majority of children had no positive family history of DDH. Our findings are consistent with previous studies conducted in China in 2017 on DDH, which have also found a higher prevalence of the condition in females than males [12]. The higher incidence in females may be due to hormonal and anatomical factors, as also depicted in a study published in ISRN Orthopedics [13]. Additionally, bilateral dislocation has been reported as the most common presentation in other studies as well, just like a study published in 2012, showing similar results about the presentation of patients in DDH [14].

In terms of management, our study found that operative management was the primary approach for DDH. This is consistent with current treatment guidelines, which recommend surgical intervention for cases that do not respond to non-operative methods [15]. Surgical intervention is recommended for DDH when non-surgical treatments are not effective in stabilizing the hip joint. The goal of surgery is to reposition the femoral head and restore normal anatomy to prevent long-term complications such as pain, difficulty walking, and arthritis. However, other studies conducted in Pakistan, have reported non-operative approaches to be effective as well, particularly in mild cases [16]. This is because they are less invasive and have fewer risks than surgical interventions.

The majority of children with DDH had no positive family history of the condition. This finding is consistent with previous studies published in StatPearls, which have found that the majority of DDH cases occur spontaneously, without a family history [17]. This may be due to the reason that DDH can be caused by a combination of genetic and environmental factors.

Based on the information presented in Table 2, it appears that the majority of children diagnosed with DDH had a normal nature of pregnancy. This finding is consistent with the results of other studies, which have also found that the incidence of DDH is not significantly associated with the nature of pregnancy [18]. The incidence of DDH is not significantly associated with the nature of pregnancy because the development of the hip joint occurs during fetal life and is influenced by factors that are largely independent of the mother’s pregnancy. However, it is noteworthy that 14.2% of cases had complications during pregnancy, with oligohydramnios being the most common complication. This discovery is in line with earlier research studies, which have found that oligohydramnios is associated with an increased risk of DDH [19]. Possibly it could be due to the reduction in the amount of space available for the developing fetus to move and grow.

In terms of delivery parameters, it is notable that C-section delivery was more common than vaginal delivery, with the breech presentation being present in 30.0% of cases. These findings are consistent with other studies that have found breech presentation is associated with an increased risk of DDH [2]. The possible reason for that is breech position can put additional stress on the hip joint during delivery. Additionally, the finding that delayed placental delivery was the most common complication during delivery is consistent with previous studies conducted in Australia, which have also found that delayed placental delivery is associated with an increased risk of DDH [20]. Delayed placental delivery is associated with an increased risk of DDH because it can lead to a decrease in blood flow to the baby’s developing hip joint.

Regarding maternal diseases, it is notable that a small percentage of mothers had endocrine diseases. These results align with previous research studies that have found that endocrine diseases are associated with an increased risk of DDH [21]. This may be due to the reason that thyroid hormones are important for the development of the hip joint.

The finding that a positive family history of DDH was reported in more than one-third of cases accords with preceding research studies, that have found that a positive family history in parents, is a significant risk factor for DDH [4]. As there is a higher incidence of DDH in families with a history of the condition, suggesting that genetic factors may play a role in its development. Genetic mutations or variations may affect the formation and development of the hip joint, leading to an increased risk of DDH.

Our study aimed to investigate the association between these factors and complications during pregnancy using Fischer’s exact test. The study included 120 women, of which 85.8% had no complications during delivery, 0.8% had an unknown status, and 13.3% experienced complications.

The results of our study regarding the mode of delivery showed that 80.8% of women who delivered via C-section had no complications, and 19.2% had complications. Among women who delivered vaginally, 89.7% had no complications, 1.5% had an unknown status, and 8.8% experienced complications. However, the difference in complication rates between the two modes of delivery was not statistically significant. These results correspond with earlier research studies that reported no significant difference in maternal morbidity or mortality rates between planned C-section and planned vaginal deliveries [22]. C-section delivery is associated with a higher risk of maternal morbidity, such as postoperative infection, blood loss, and longer hospital stays. However, planned C-section delivery may be necessary in cases where vaginal delivery poses a higher risk to the mother or baby, such as in cases of placenta previa, breech presentation, or certain medical conditions. 

Regarding presentation during delivery, the study found that 86.1% of women with breech presentation had no complications and 13.9% experienced complications. Among women with a cephalic presentation, 87.2% had no complications and 12.8% experienced complications. However, the difference in complication rates between the two groups was not statistically significant (p = 0.094). Similarly, prior studies reported no significant difference in the risk of complications between breech and cephalic presentations [23]. But the likelihood and severity of these complications can vary depending on various factors such as the baby’s size, position, and the mother’s health status.

## Conclusions

DDH is a significant health issue that affects newborns and can lead to long-term mobility problems and discomfort. A positive family history of DDH is a significant risk factor for the condition. Early detection and treatment of DDH are crucial to prevent long-term hip dysplasia and arthritis. The study highlights the importance of family history as a risk factor for DDH and emphasizes the need for screening programs in families with a history of the condition.

Limitations

Sample Size

The study has a relatively small sample size of 120 children, which may limit the generalizability of the findings.

Selection Bias

As the study was conducted in an urban setting, the results may not be representative of the general population. Additionally, the sample was likely selected from hospital records, which may have excluded children who were not diagnosed or treated for DDH.

Data Collection

The study relied on retrospective medical record reviews, which may have limited the completeness and accuracy of the data collected. There may have been missing data or errors in the medical records, which could have affected the results.

Confounding Factors

The study did not investigate other factors that may have contributed to the development of DDH, such as environmental factors or genetic predisposition. Without controlling for these factors, it is difficult to draw definitive conclusions about the associations found in the study.

Treatment Bias

The study found that operative management was the most common management strategy for DDH. However, the study did not investigate the reasons why some children received non-operative management or why some received both operative and non-operative management. Without this information, it is difficult to assess the appropriateness of the treatment decisions made.
